# Healthcare workers’ perceptions of an organizational quality assurance program implemented in a resource-limited setting: a qualitative study

**DOI:** 10.15171/hpp.2018.24

**Published:** 2018-07-07

**Authors:** Hiroko Henker, Shivani Fox-Lewis, Navy Tep, Dary Vanna, Sreymom Pol, Claudia Turner

**Affiliations:** ^1^Graduate School of Public Health, University of Pittsburgh, Pittsburgh, USA; ^2^Cambodia Oxford Medical Research Unit, Siem Reap, Kingdom of Cambodia; ^3^Centre for Tropical Medicine & Global Health, Nuffield Department of Medicine, University of Oxford, Oxford, UK; ^4^Angkor Hospital for Children, Siem Reap, Kingdom of Cambodia; ^5^Mahidol Oxford Tropical Medicine Research Unit, Faculty of Tropical Medicine, Mahidol University, Bangkok, Thailand

**Keywords:** quality assurance, Quality of care, Provider perception, Resource-limited setting

## Abstract

**Background:**There is increasing awareness of the need to implement quality assurance programs in developing countries. Healthcare staff are the primary drivers of improving the quality of care,but little is known about how they perceive quality assurance programs in resource-limited settings. This study aims to evaluate healthcare workers’ perceptions of the organizational quality assurance program (OQA) at Angkor Hospital for Children (AHC), Cambodia. The OQA involves regular data collection and monitoring of quality indicators, to assess whether agreed quality standards are being met.

**Methods: ** This qualitative study consisted of four focus group discussions (FGDs) with 29 hospital staff (convenience sampling) from medical, nursing and non-medical departments. Staff members’ understanding of quality assurance and perceptions of the strengths and weaknesses of the OQA were explored. Thematic content analysis was used to identify key themes.

**Results:** Participants emphasized that quality indicators must include physical and psychological well-being. Strengths of the OQA included shared understanding amongst all groups of participants of its goals, committed leadership, that it was locally-relevant and that target indicators were developed from a "ground-up" approach. On-going challenges included that there was a gap in understanding of the OQA processes and overall running of the OQA across the organization between managers and staff.

**Conclusion: ** The introduction of the OQA at AHC has been well-received by staff members.Overall, the program is perceived to be valuable. Healthcare provision in resource-limited settings increasingly needs to demonstrate quality assurance. The model of OQA developed at AHC is one way to achieve this.

## Introduction

Quality assurance is defined as “activities that are carried out to set standards and to maintain and improve performance so that the care provided is as effective and safe as possible.”^1^ In developing countries, the focus of healthcare provision has traditionally been on the delivery of healthcare services. Increasingly, there is a shift in developing countries towards quality assessment and assurance of healthcare services provided.^2^ In developing countries there may be a gap between current practice and best practice, highlighting the potential of quality assurance programs.^3^ There is an increasing evidence base for the feasibility and efficacy of such programs in developing countries. However, much of the evidence in healthcare has come from vertical programs involving single units or specific areas, focusing on targeted outcomes.^3^ There is a lack of reported data on the development of healthcare organization-wide quality assurance programs.

Quality assurance programs must themselves be evaluated for success, to determine whether they meet their desired goals. One aspect of measuring the success of quality assurance programs is to determine the perceptions of the program, from the perspective of key stakeholders.^4^

Hospital employees (clinical and non-clinical) play essential roles in quality assurance programs.^5^ Their perceptions of the programs are important aspects of determining program success.^4^ There is little reported literature exploring hospital employees’ perceptions about quality assurance programs and their success in resource-limited settings.

### Organizational quality assurance program at Angkor Hospital for Children

**Figure 1:**
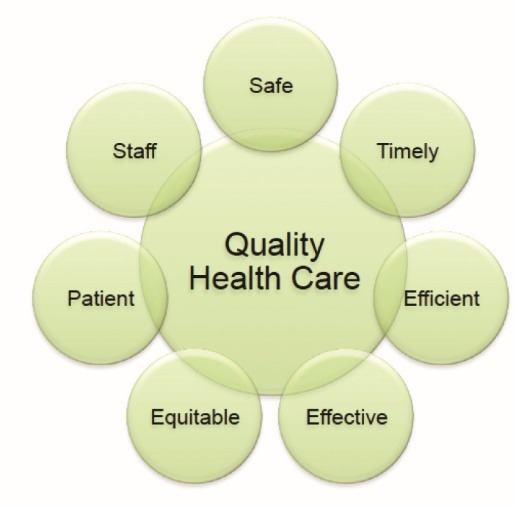
The conceptual schema for the organizational quality assurance program at Angkor Hospital for Children.

The Angkor Hospital for Children (AHC) organizational quality assurance program (OQA) was launched in January 2016, with the aim of establishing measurable quality indicators and standards, and ensuring a coordinated approach to the delivery of high quality care. The program was based on a model devised by the Institute of Medicine and uses the pneumonic “STEEEPS” (Safe, Timely, Efficient, Effective, Equitable, Patient and Staff) to focus key priorities of the program (Figure 1).^6^

The OQA program consists of 3 main activities: on-going data collection of routine hospital activities (number of inpatients and outpatients, bed occupancy rate, length of stay); activities and data collection for specific targets areas (Box 1); and the establishment of the OQA committee. The OQA committee is responsible for implementing the OQA. It is made up of 3 working groups (medical, nursing and non-medical). Each working group comprises a number of units (e.g. nursing surgical unit, medical surgical unit etc). Each unit was required to develop one or two quality indicators, called key performance indicators (KPIs). Examples of KPIs are illustrated in Box 2.

The OQA, therefore, consists of organization-led organization-wide targets (Box 1) in addition to unit-driven targets (KPIs). Data collected on the OQA activities is reported regularly to the hospital data management team. They in turn produce reports for the AHC executive committee. The process of implementation and on-going running of the OQA is shown in Box 3.

Based on the abovementioned background, this study aimed to explore the perceptions of hospital staff on the OQA at a Cambodian pediatric hospital. This study explored the perceptions of hospital staff, with regard to strengths and weaknesses of the OQA, and whether staff felt that the OQA appropriately met the needs for quality improvement for the setting.

## Materials and Methods

### Study design 

This qualitative study used focus group discussions (FGDs) to explore the perceptions of hospital staff regarding the OQA at AHC, to determine which aspects of the program have been successful and where improvements could be made.

### Participants and procedures

*Setting*

AHC was established in 1999 and is a non-governmental, non-profit pediatric referral hospital in Siem Reap, Cambodia. The hospital provides free health care to all children under 16 years of age, and includes outpatient, inpatient and community outreach services. AHC’s vision statement is that “all Cambodian children have access to high quality, compassionate care wherever they live and whatever their ability to pay.”^7^

*Sampling*

Participants in the FGDs were selected by convenience sampling within 3 employee groups at AHC (medical, nursing and non-medical), and 3 different roles in the OQA (department manager, OQA committee member, and non-committee member). Sign-up sheets were put up on bulletin boards at different departments for potential participants to indicate their interest. Senior management staff also announced the study at staff meetings. Potential participants were approached by the study team and given detailed information about the study. Written informed consent for participation in the study was obtained prior to participation.

A total of 29 participants took part in 4 FGDs. The details of participants’ demographics are presented in Table 1. The median duration of the FGD was 36 minutes (range 33-37 minutes).

### Data collection

Four FGDs were conducted in July 2017. The discussions were conducted primarily in English. If they preferred to, participants could express their views in Khmer (national language of Cambodia), with translation offered by Khmer-speaking members of the study group. An iterative topic guide was used to facilitate the discussions, exploring participants’ understanding of the OQA and their views on its strengths and weaknesses. The FGDs were audio-recorded, and field notes were made by the study team. After each FGD, the study team discussed the findings and revised the topic guide as needed. Data collection continued until data saturation was reached.

### Data analysis and trustworthiness

The audio-recordings were transcribed, translated (where needed) and checked. The transcripts and field notes were imported into NVivo version 11 (QSR international, Victoria, Australia) to aid thematic content analysis of the data. An inductive data-driven approach, with no prior assumptions, was applied in order to identify key themes within the data.^8^ Key themes were discussed, revised and agreed upon within the study group.

## Results

The key themes emerging from the data are presented below; participants’ understanding of the OQA, strengths of the OQA and weaknesses of the OQA. Within “strengths of the OQA” the subthemes are: appropriateness of the OQA, measurement of the quality of care provided, standardizing and improving practice and success of the OQA. Within “weaknesses of the OQA” the subthemes are: difficulty in assessing compassionate care, additional work of the OQA and strain on resources.

### Participants’ understanding of the OQA

Managers stated that the OQA was an overarching mechanism to improve and strengthen the quality of care provided by AHC. They described that their activities for the OQA were to establish plans to address quality issues and to coordinate teamwork between and within departments to achieve the goals of the OQA.

In contrast, some participants from the OQA committee and non-committee members saw the OQA as synonymous with the KPIs. They described the OQA as consisting of activities to find and improve weak points and mistakes in the quality of care provided within their units. These participants did not view the OQA as a wider quality assurance endeavor, viewing it much more as local departmental activities. As such, they felt that the OQA program has succeeded because the KPIs that they were currently working on had reached their targets.

*“I think our activities are good based on the KPI results. Because the KPI result was better than our expectation.”* (Medical, Non-committee member)

A minority of participants had very limited understanding of the OQA, for example, they did not know what the abbreviation “OQA” stood for. One participant was not familiar with either the OQA or the KPI.

*“Actually, I don’t know about OQA, I don’t know KPI.”* (Medical, Non-committee member)

Managers acknowledged this gap in the understanding of the OQA across hospital staff. One manager emphasized the importance of sharing information and the involvement of all hospital staff in the OQA program, in order to address this gap.

*“This is the gap, which we could not reach everybody in the hospital. All should be involved. [...]All information about the OQA should be provided to all staff. We should be responsible for this.*” (Non-medical, Manager)

### Strengths of the OQA

*Appropriateness of the OQA*

Participants reported that providing good quality care involves several dimensions. For many participants it meant providing good health results to patients. Some participants added that patients should reach this goal without suffering harm due to the treatments provided. Participants also mentioned that the psychological well-being of patients and their families was important.

*“I think the quality of care, if I had to define it in one phrase, is combining everything to make patients feel comfortable, to make the families of patients feel comfortable, and to help patients get better from sickness and make them feel happy as well.”* (Non-medical, Manager)

Participants recognized that the target indicators within the OQA aimed to assess these various dimensions.

Furthermore, several participants pointed out that evaluation of the quality of care delivered should be done in a locally specific context, as is the case with the OQA, which was developed specifically for the context of AHC.

*“The quality of care also depends on the place, the hospital, or the country where those services are delivered”.* (Medical, Committee member)

*Measurement of the quality of care provided*

Participant said that they were proud of the quality of care provided at AHC but did not know how they could measure this or compare it to previous years, or to other hospitals. Participant perceived the OQA as filling this gap, and providing demonstrable measures of the high quality of care provided at AHC.

*“Before we implemented the OQA program, we knew that AHC provides high quality of care. But how did we know it was high quality? There were no measurements. KPIs and OQA are to measure quality so that you can compare to the other facilities and the other countries. So in this way, we can know that we provide high quality care, because we can see the results of the KPIs.”* (Medical, Non-committee member)

Participants placed great value on providing high quality care, despite the resource constraints they practiced within. Participants were clear that a lack of resources should not be a deterrent to providing high quality care within the means available.

*“We know how to treat patients, but we have the lack of resources. So what we need to do is just set some goals that we can achieve at this point. So it may not really be the very best treatments, but we need to focus on what we can do with the resources that we have.”* (Nurse, Non-committee member)

*Standardizing and improving practice*

Participants reported that the OQA provided a method for standardizing hospital practices, and maintaining them at an agreed high standard.

*“Because the standard that we set in our hospital is like the international standard. If we apply and provide our services following those, patients can get high quality care”* (Non-medical, Non-committee member)

Participants reported that this resulted in improved knowledge and skills, therefore resulting in improved practice.

*“The nurses need to train to have the knowledge to provide care to patients, skills, and competencies. So those all are necessary qualities. So for high quality care nurses need to be trained and increase their knowledge to provide good quality care.”* (Nurse, Non-committee member)

The participants also pointed out that the KPIs increased their awareness of their performance and played a role as a reminder of the agreed standards. For example, one of the KPIs for the nursing department was the standardized procedure for suctioning (suctioning is used to remove excessive oral fluid secretions from patients). The indicator assessed how accurately nurses performed suctioning based on the procedural guideline. One of nurse participants mentioned that the KPI reminded nurses of the necessary steps they were expected to perform during suctioning.

*“It is like a reminder for the member to suction correctly based on the procedure… When we apply this KPI at the unit, the staff who take care of the patient will be aware that there are people watching him/her. So they know that they have to know all the steps of suctioning.”* (Nurse, Non-committee member)

Standardization in non-medical hospital services was also considered to be crucial. Participants from non-medical departments mentioned that even though it does not have a direct impact on patients’ health, organizational well-being allows the smooth-running of hospital services, as well as the satisfaction of hospital staff.

*Success of the OQA*

The majority of participants reported that the implementation of the OQA had been successful. Managers pointed out that since the OQA has only been operational for one year, it is too soon to determine its impact on healthcare provision. However, they were pleased with its operational progress and implementation.

“*Because everyone knows that when talking about quality it is not a ‘one-day’ task to achieve improvement. That’s why we continue working on quality improvement.”* (Nurse, Manager)

### Weaknesses of the OQA

*Difficulty in assessing compassionate care*

Participants reported that not all aspects of the OQA worked as well as others. One aspect in particular that proved challenging, according to participants, was measuring compassionate care.

Providing compassionate care is a key principle underlying AHC’s activities. For almost all participants, the most important factor in delivering good quality care was compassion. Participants described that providing compassionate care meant to treat a patient as their own child. Participants saw good communication and empathy-driven staff motivation as components of delivering compassionate care:

*“When doctors have good communication with parents and explain the problem to parents so that they can understand, then they accept our explanation, which makes them feel comfortable.”* (Medical, Manager)

When asked how to measure compassionate care, the participants listed several methods, one of which was the patient satisfaction survey that had been done by the Young Persons Advisory Group at AHC.^9^ One participant mentioned regular patient satisfaction surveys could be used to see progress in providing compassionate care. Other suggestions included obtaining feedback from all stakeholders (e.g. patients, families, communities and the government), peer evaluation, observation of patient behaviors, and anecdotal episodes.

*“I have heard from my friends, some friends who took their children to AHC, they are happy to come to AHC because doctors and nurses are providing compassionate care.”* (Non-medical, Manager)

However, some participants disagreed with the measurability of compassionate care, claiming that results from such measurement methods would not be reliable because the definition of compassionate care was subjective and because responses from patients and families may be biased because they had received free services.

*“Because we cannot ask the patient how is our treatment here because its treatment is free. Patients would have difficulty in saying that a doctor is bad. I think this is not an accurate way to measure compassionate care.”* (Medical, Non-committee member)

### Additional work of the OQA

The main challenge for the OQA committee members and non-committee members was that performing activities for the KPIs sometimes represented additional work and interfered with their on-going routine duties.

*“So the staff do not only work for the OQA activities. They have a lot of things to do, their main job to do.”* (Nurse, Committee member)

This was reported more by medical and nursing participants rather than non-medical participants. However, one manager described no challenges in his department because the tasks for the KPIs did not require any additional work than routine tasks.

*“The reason is the tasks. The tasks for the OQA activities are similar to the tasks we are doing daily.”* (Non-medical, Committee member)

Ground staff also felt it was difficult to follow the standardized steps of procedures set out in the KPIs because of interruptions due to emergency cases and a lack of human resources. In addition, they listed insufficient equipment supply as another reason that they could not follow the standards.

*“It is really difficult because we work with patients, we use supplies and equipment, but sometimes the equipment is not working properly.”* (Nurse, Non-committee member)

### Strain on resources

The OQA committee members said that their activities for the KPIs were not well coordinated because the committee members could not assemble or discuss easily due to scheduling difficulties. They felt this impacted their OQA activities and meant that the OQA was not implemented as well as it could be. They could not manage supervision of the KPIs in a timely manner, delaying evaluation of the KPIs. Other OQA committee members said that they had excessive demands on their time and felt that more human resources were needed to achieve the OQA goals.

*“We set the goal of two staff to prepare medicines for dispensing. But now we have more jobs, such as preparing chemotherapy, drug mixing, and only one staff available to prepare medications. That’s why the KPI result was decreased.” (Medical, Committee member).*

## Discussion

This study explored hospital staff members’ perceptions of the OQA, its strengths and weaknesses, at AHC, a resource-limited healthcare setting in Cambodia.

The majority of participants were familiar with the OQA and their role within it. Managers had a good understanding of the structure of the OQA; some other staff viewed it as synonymous with KPIs. Managers recognized there was a need to improve communication with all staff around OQA activities.

One major strength of the OQA at AHC was the shared belief in the need for the program and its value, at all levels of leadership and staff. This resulted in greater ‘buy in’ to it at all levels. As shown in this study, all participants believed in providing high quality compassionate care, echoing AHC’s vision. Sharing this common goal was fundamental to the success of the OQA.

A key feature of the OQA was its strong leadership. This study found that the managers were aware of their roles as leaders and have taken the responsibility for running the OQA throughout the hospital. From their point of view, the OQA has been smoothly implemented, although at this early-stage evaluation it is not yet possible to comment on its impact on healthcare outcomes.

Key strengths of the OQA included that it was seen as contextually appropriate, and consisting of indicators that reflect how participants viewed “high quality care”. The ability to measure the quality of care provided was important. Staff could visualize their efforts towards the improvement in the quality of care provided, which could encourage their involvement and commitment to the OQA.

Fundamental to this commitment was that the KPIs were developed from a ‘ground-up’ approach, with each department identifying areas to target and designing activities to address them. The OQA committee members worked with ground staff to identify areas that needed to be improved. Participation in decision-making is associated with job commitment^10^; therefore, this manner of developing the KPIs could increase hospital staff members’ commitment towards completing the KPIs.

Participants also reported that the OQA allowed more standardization in practice, and improved practice, because the KPIs reminded staff of the necessary procedures to follow. Overall, all groups of participants viewed the OQA as a successfully implemented program.

Previous studies have reported that a lack of human resources was the main barrier to providing high quality of care, regardless of economic conditions.^11,12^ Whilst this was mentioned as a barrier, in this study participants felt that it was very possible to deliver high quality care within the constraints of the resources available. For participants in this study, the main barrier to implementation of the OQA was difficulty in the operational processes of the OQA. Participants valued the OQA, but in some instances found that it interfered with their routine tasks. Further streamlining of processes to measure, record and report OQA targets, within and between departments would improve the smooth-running of the program.

Participants also raised the point that measuring compassionate care is difficult. Whilst patient satisfaction surveys are the current standard for this, participants noted that they might not be truly indicative of patients’ views. The concerns regarding the validity of the survey included that the provision of free healthcare may results in biased responses from patients and their caregivers.

Participants did not feel that the OQA incorporated the assessment of patients’ psychological wellbeing at present. One reason for this may be that the majority of staff (not involved with the development of the OQA) saw it as synonymous with KPIs. Therefore, since the KPIs do not include targets to measure patient satisfaction or compassionate care, these staff felt that further activities were needed. However, one of the OQA activities does include the patient satisfaction survey conducted by the Young Persons Advisory Group.^9^ Participants also mentioned the need to have greater patient and community engagement, which is also part of AHC’s activities.^9^ These findings highlight that whilst conducting activities to improve the quality of care in a holistic manner is essential, equally important is communicating this to all staff. In this way, hospital staff can better appreciate the overarching reach of the OQA.

As discussed above, all staff share a common understanding of the goals of the OQA. However, as noted in the above example of measuring compassionate care, all staff do not have a complete understanding of the OQA itself. There appeared to be a gap in understanding, in which managers understand the activities of the OQA and see it as the way to achieve the AHC’s goals. However, for the majority of non-committee staff, the OQA means KPIs. They have a fragmented understanding of KPIs across the organization and do not see the overarching activities of the OQA as a whole. Improved communication of the whole OQA program and its targets and results could improve staff understanding of it.

**Figure 2:**
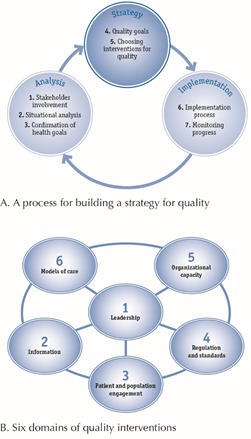
The framework for quality assurance interventions suggested by WHO.^13^
Reproduced from *quality of care: a process for making strategic choices in health systems 2006.*

The aspects of the OQA discussed fit into the framework proposed by the World Health Organization (WHO). The WHO suggested that “a process for building a strategy for quality” is a circulatory process, including seven elements which fall into 3 categories: analysis, strategy, and implementation (Figure 2).^13^ This framework depicts the processes involved in the design and implementation of the OQA, from the initial situational analysis involving key stakeholders and defining standards together, to choosing interventions and assigning appropriate targets. The onward implementation and monitoring is an on-going process. In this resource-limited setting it was feasible to implement such a strategy, using the key concepts of leadership, recording reliable data on indicators and the sharing of information, employing regulations and standards, and doing so within the locally-specific models of care and the organizational capacity.

There are several limitations of this study. This study was conducted at a single non-governmental site in Cambodia. The context of the OQA here may, therefore, be different to other facilities. However, the importance of a locally-contextualized OQA has been discussed, and the findings of this study, with regard to the key strengths and weaknesses of an OQA, would be applicable to a range of settings. Readers could implement and modify their OQA programs based on the successes and difficulties discussed in this study. Study participants were recruited by convenience sampling, which may have resulted in some participants who wished to be involved not being able to do so. This pragmatic sampling method was necessary in order to respect participants’ duties to the hospital.

## Conclusion

The AHC OQA has been implemented for one and a half years. Although it is too soon to evaluate the impact of the OQA on the quality of care provided by AHC, participants viewed its implementation as successful. An OQA is seen as necessary in standardizing, improving and measuring the quality of care provided. Improved communication and streamlining of OQA activities are necessary challenges to address. The incorporation of both management-led and ground-up development of quality improvement targets is essential in ensuring the relevance of, and staff commitment to, the OQA.

## Ethical approval

The study was approved by the Institutional Review Board of Angkor Hospital for Children, in Siem Reap, Cambodia (0870/17 AHC), and by the Institutional Review Board of the University of Pittsburgh, in Pittsburgh, Pennsylvania, USA (PRO107505312). After a full explanation of the study was provided, and prior to commencing participation, all participants provided written informed consent. This study was conducted in accordance with the principles of the Declaration of Helsinki and its amendments.

## Competing interests

The authors declare that they have no competing interests.

## Authors’ contributions

HH conceived the study, collected the data, conducted the analysis and drafted the manuscript. SFL interpreted data and wrote the manuscript. CT oversaw the whole study process, designed the study, analyzed and interpreted data, and critically reviewed the manuscript. SP collected, translated, analyzed and interpreted data, and reviewed the manuscript. DV recruited the study participants, organized the focus group discussions, collected, translated, analyzed and interpreted data, and reviewed the manuscript. NT arranged the recruitment of the study participants and reviewed the manuscript. All authors read and approved the final manuscript.

## Acknowledgements

The authors would like to extend their gratitude to the staff at Angkor Hospital for Children.

**Box 1:** Examples of organization-wide indicators in the OQA

**Examples of organization-wide indicators**
1. Medication errors
2. Infection prevention and control
a. Hand hygiene surveillance
b. Healthcare associated infections
3. In-hospital deaths
4. Patient satisfaction
5. Staff accidents on site
6. Waiting times
7. Patients leaving without being seen by a healthcare worker
8. Number of patients turned away because of limited doctor numbers
9. Number of patients sent to another facility due to lack of available beds at AHC
10. Discharge planning
OQA: Organizational quality assurance program, AHC: Angkor Hospital for Children.

**Box 2:** Examples of key performance indicators

**Examples of key performance indicators**
Medical
• The number of patients given oral health education
• The effectiveness of interventions at the intensive care department
• The number of radiology reports produced on time
Nursing
• Waiting time in the outpatient department
• Infant body temperature measurement
• Procedural errors using standard guidelines (e.g. suction)
Non-medical
• Financial indicators
• The number of expired supplies
• Employees’ turnover rate

**Box 3:** The process of implementing the OQA at AHC

**Process of implementing the OQA**
1. Inclusion as an organizational strategic priority
2. CEO led
3. Literature review
4. Review of other organizational models
5. Model and objectives drafted by CEO and discussed by executive committee
6. Name discussed and given to program
7. Presentation to the Board of Directors for approval
8. Organization wide meeting (representatives from all units) to discuss quality, its meaning, and measurement
9. Formation of the OQA committee and working groups
10. Monthly reports of routine hospital activities and specifically targeted areas discussed with senior management to identify abnormal or worrying trends and action plans devised and carried out
OQA: Organizational quality assurance program, AHC: Angkor Hospital for Children, CEO: Chief executive officer.

**Table 1:** Demographics of participants

**Variables**	**N = 29**
Age, No. (%)	
21-25	1 (3.4)
26-30	4 (13.8)
31-35	11 (37.9)
36-40	5 (17.2)
41-45	5 (17.2)
46-50	2 (6.9)
Unknown	1 (3.4)
Gender, No. (%)	
Male	16 (55.2)
Female	13 (44.8)
Department, No. (%)	
Medical	8 (27.6)
Nursing	12 (41.4)
Non-medical	9 (31.0)
Length of service, No. (%)	
Less than 4 years	4 (13.8)
4 to 6 years	8 (27.6)
7 to 9 years	2 (2.9)
10 to 12 years	6 (20.7)
13 to 15 years	1 (3.4)
More than 15 years	4 (13.8)
OQA committee membership, No. (%)	
Manager	8 (27.6)
Member	6 (20.7)
Non-member	15 (51.7)

OQA, Organizational quality assurance program.

## References

[1] Brown LDP, Franco LM, Rafeh N, Hatzell T. Quality assurance of health care in developing countries: Quality assurance methodology refinement series. Bethesda, Maryland: Quality Assurance Project; 1993.

[2] Peabody JW, Taguiwalo MM, Robalino DA, Frenk J. Improving the quality of care in developing countries. In: Jamison DT, Breman JG, Measham AR, eds. Disease Control Priorities in Developing Countries. 2nd ed. Washington, DC: World Bank; 2006. p. 1293-308. 21250362

[3] Leatherman S, Ferris TG, Berwick D, Omaswa F, Crisp N (2010). The role of quality improvement in strengthening health systems in developing countries. Int J Qual Health Care.

[4] Kaplan HC, Brady PW, Dritz MC, Hooper DK, Linam WM, Froehle CM (2010). The influence of context on quality improvement success in health care: a systematic review of the literature. Milbank Q.

[5] Health Resources and Services Administration. Quality Improvement. U.S. Department of Health and Human Services; 2011 https://www.hrsa.gov/quality/toolbox/methodology/qualityimprovement/. Accessed August 9, 2017.

[6] Institute of Medicine Committee on Quality of Health Care in America. Crossing the Quality Chasm: A New Health System for the 21st Century. Washington, DC: National Academies Press; 2001. doi:10.17226/10027.

[7] Angkor Hospital for Children. http://angkorhospital.org/. Accessed July 29, 2017.

[8] Chandler CIR, Reynolds J, Palmer JJ, Hutchinson E. ACT Consortium Guidance: Qualitative methods for international health intervention research. London: London School of Hygiene & Tropical Medicine; 2013.

[9] Pol S, Fox-Lewis S, Cheah PY, Turner C (2017). “Know your audience”: A hospital community engagement programme in a non-profit paediatric hospital in Cambodia. PloS One.

[10] Witt LA, Andrews MC, Kacmar KM (2000). The role of participation in decision-making in the organizational politics-job satisfaction relationship. Hum Relat.

[11] Aiken LH, Sermeus W, Van den Heede K, Sloane DM, Busse R, McKee M (2012). Patient safety, satisfaction, and quality of hospital care: cross sectional surveys of nurses and patients in 12 countries in Europe and the United States. BMJ.

[12] Islam F, Rahman A, Halim A, Eriksson C, Rahman F, Dalal K (2015). Perceptions of health care providers and patients on quality of care in maternal and neonatal health in fourteen Bangladesh government healthcare facilities: a mixed-method study. BMC Health Serv Res.

[13] World Health Organization. Quality of care: a process for making strategic choices in health systems. Geneva: WHO; 2006.

